# Laparoscopic colopexy for neo-left colonic volvulus 10 years after anterior resection

**DOI:** 10.1093/jscr/rjaa555

**Published:** 2020-12-31

**Authors:** Roy Huynh, Mifanwy Reece, David Mansouri, Thuy-My Nguyen, Anil Keshava

**Affiliations:** Department of Colorectal Surgery, University of Sydney, Concord Repatriation General Hospital, Sydney, New South Wales, Australia; Faculty of Medicine, University of New South Wales, Sydney, New South Wales, Australia; Department of Colorectal Surgery, University of Sydney, Concord Repatriation General Hospital, Sydney, New South Wales, Australia; Department of Colorectal Surgery, University of Sydney, Concord Repatriation General Hospital, Sydney, New South Wales, Australia; Department of Colorectal Surgery, University of Sydney, Concord Repatriation General Hospital, Sydney, New South Wales, Australia; Department of Colorectal Surgery, University of Sydney, Concord Repatriation General Hospital, Sydney, New South Wales, Australia; Faculty of Medicine and Health Sciences, Macquarie University, Sydney, New South Wales, Australia

## Abstract

Recurrent neo-left colonic volvulus is a rare complication following anterior resection. The conventional approach to treating recurrent volvulus is a large bowel resection with anastomosis or colostomy formation after successful endoscopic decompression. However, in elderly and comorbid patients, this can result in significant morbidity or mortality. Laparoscopic colopexy is a less invasive alternative that has not been previously reported for the treatment of neo-left colonic volvulus. We describe a case of an 86-year-old male who presented with recurrent neo-left colonic volvulus 10 years post-laparoscopic anterior resection for cancer. A laparoscopic colopexy was performed to resolve the volvulus and prevent future recurrence. Interrupted prolene sutures were used to fix the neo-left colon to the posterior stomach and the left lateral abdominal wall. The patient had an uncomplicated postoperative recovery and was discharged 6 days after surgery. He was well at 6 months follow-up.

## INTRODUCTION

Recurrent volvulus of the neo-left colon is a rare complication following laparoscopic anterior resection [1]. It occurs due to torsion of the redundant left colon formed following mobilization during the index operation. Pre-disposing factors such as constipation and neurological disease can accentuate the presentation of recurrent volvulus. The conventional approach to managing recurrent volvulus is resection of the redundant colon with anastomosis or end colostomy following successful endoscopic decompression [2]. However, in elderly and comorbid patients, major colonic resection can result in significant morbidity or mortality. Percutaneous endoscopic colopexy (PEC) is a less invasive alternative to surgery and involves the use of fixation tubes to affix the colon to the abdominal wall [3]. While viable, this approach requires transmural penetration of the abdominal and colonic wall and is associated with complications such as skin and soft tissue infection (including necrotising fasciitis), fecal leakage and peritonitis. Furthermore, the fixation tubes can be unintentionally pulled out by patients with cognitive impairment [4]. The use of PEC tubes following prior left colonic resection carries additional risks due to the altered anatomy. Laparoscopic colopexy is less invasive than colectomy and does not share the same risk profile as PEC. In this paper, we report a case of an 86-year-old male with cognitive impairment and Parkinson’s disease who underwent a laparoscopic colopexy for a recurrent volvulus in the context of a prior laparoscopic anterior resection for cancer.

## CASE REPORT

An 86-year-old male with cognitive impairment and Parkinson’s disease presented with recurrent volvulus of the neo-left colon requiring numerous endoscopic decompressions. The patient had undergone a curative laparoscopic low anterior resection 10 years prior for a rectal adenocarcinoma.

**Figure 1:**
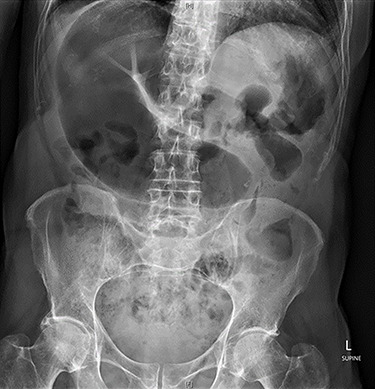
Abdominal X-ray on presentation showing a distended loop of colon with air-fluid levels.

**Figure 2:**
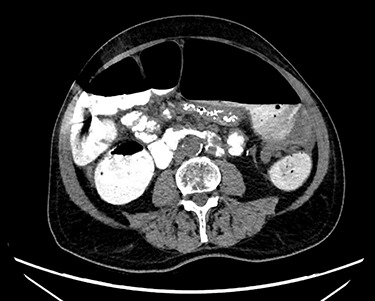
CT scan demonstrating a dilated colonic loop with air-fluid levels and swirling of the mesentery, indicative of a large bowel obstruction secondary to a volvulus.

**Figure 3:**
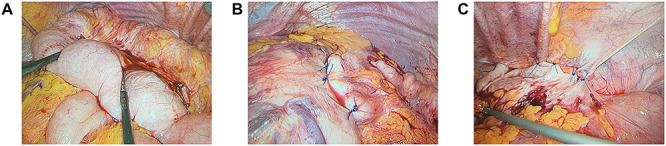
(**A**) Neo-left colonic volvulus forming an alpha-type loop; (**B**) pexy of the neo-left colon to the posterior stomach wall using interrupted prolene sutures; (**C**) pexy of the neo-left colon to the left lateral abdominal wall using interrupted prolene sutures

Abdominal X-ray revealed a grossly distended loop of colon with air-fluid levels, consistent with a large bowel obstruction (Fig. 1). A computed tomography (CT) scan demonstrated two transition points in the left colon with swirling of the mesentery, indicative of volvulus (Fig. 2). Following successful endoscopic decompression, the patient proceeded to laparoscopy and colopexy. The previous laparoscopic port sites were utilized. On table colonoscopy was performed during laparoscopy to allow delineation and decompression of the volvulus. As seen in Fig. 3A, the neo-left colon travelled behind the transverse colon mesentery and formed an alpha-type loop. Adhesions fusing the transverse colon mesentery to the neo-left colon were divided to mobilize the colon and to assist in devolving the bowel (Video 1). Interrupted polypropylene sutures were then used to fix the neo-left colon to the posterior gastric wall (Fig. 3B) and the left lateral abdominal wall (Fig. 3C). The patient’s bowels opened on postoperative Day 3 and he was discharged on postoperative Day 6. The patient remained well at 6 months follow-up with no further episodes of volvulus.

## DISCUSSION

Large bowel volvulus is an uncommon cause of obstruction and occurs when a redundant loop of colon twists upon its mesenteric attachment. In most cases, the long redundant colon is a result of chronic constipation, where prolonged colonic distension due to fecal loading can result in elongation [5]. Laparoscopic surgery mitigates the formation of intraperitoneal adhesions and this can predispose to redundancy and volvulus of previously mobilized colon [6]. A systematic review by Toh *et al*. [1] showed that volvulus following colorectal surgery is rare and nearly all cases took place within 4 months of surgery. Our patient experienced his first volvulus 4 years after his anterior resection, suggesting that other factors such as his Parkinson’s disease (causing slow transit time) may have played a contributary role.

The primary treatment for an acute volvulus is decompression with either a rigid or flexible endoscope. This approach is initially successful in ~80% of uncomplicated cases [7]. Surgery is indicated in the acute setting if endoscopic decompression fails to relieve the volvulus or if gangrenous bowel is encountered. The recurrence rate for patients who successfully undergo endoscopic decompression has been reported to be as high as 90% [8]. However, many of these patients can be managed with repeated endoscopic decompressions. The decision to perform surgery in the same admission following resolution of acute volvulus has been advocated by some surgeons to prevent future recurrence [7].

The conventional surgical approach is to resect the redundant colon followed by an anastomosis or formation of a colostomy. In this particular case, re-resection of the left colon would be associated with high morbidity and mortality in this elderly and comorbid patient. An alternative approach reported in the literature is PEC, however the anatomic changes following previous major colorectal resection precluded the use of this technique [3, 4].

Laparoscopic colopexy is less invasive than a colectomy and theoretically has fewer complications than PEC given that neither the abdominal nor colonic wall is transmurally or persistently breached. Laparoscopic colopexy for splenic flexure volvulus has been previously reported in an infant with good result [9]. However, there have been no previous case reports on its use in adult patients or following major left sided colorectal resection. Although the recurrence rate following laparoscopic colopexy remains to be studied, a review by Rabinovici *et al*. [10] found open detorsion with caecopexy for caecal volvulus to have lower rates of complications (15% vs 52%), recurrence (13% vs 14%), and mortality (10% vs 22%) than caecostomy tube insertion. In sigmoid volvulus, colopexy is associated with 20% recurrence compared to no recurrence with bowel resection. It is however, associated with less postoperative morbidity.

In conclusion, recurrent neo-left colonic volvulus is a rare complication following anterior resection that can occur many years after surgery. Laparoscopic colopexy is a suitable technique in preventing volvulus recurrence in patients with a high estimated morbidity or mortality. It is less invasive than conventional colectomy and avoids the need for bowel resection or stoma. It also carries less risks than PEC as it does not involve transmural penetration of the abdominal or colonic wall. With an aging population, and increased utilization of minimally invasive techniques for left colonic resection, surgeons are likely to encounter elderly and frail patients with increasing frequency and should consider using laparoscopic colopexy as a new treatment strategy in patients with neo-left colonic volvulus.

## CONFLICT OF INTEREST STATEMENT

None declared.

## FUNDING

None.

## Supplementary Material

Video_1_rjaa555
